# Influence of Interlayer Temperature and Welding Sequence on the Temperature Distribution and Welding Residual Stress of the Saddle-Shaped Joint of Weldolet-Header Butt Welding

**DOI:** 10.3390/ma14205980

**Published:** 2021-10-11

**Authors:** Chunliang Mai, Xue Hu, Lixin Zhang, Bao Song, Xiongfei Zheng

**Affiliations:** 1 College of Mechanical and Electrical Engineering, Shihezi University, Shihezi 832003, China; maichunliang@stu.shzu.edu.cn (C.M.); zhlx2001329@163.com (L.Z.); songbao@hust.edu.cn (B.S.); zxf13821795839@163.com (X.Z.); 2 College of Mechanical Science and Engineering, Huazhong University of Science and Technology, Wuhan 430074, China

**Keywords:** weldolet, header, numerical simulation, saddle-shaped welded joints, welding sequence

## Abstract

In this paper, based on Simufact Welding finite element analysis software, a numerical simulation of the temperature and residual stress distribution of the weldolet-header multi-layer multi-pass welding process is carried out, and the simulation results are verified through experiments. The experimental results are in good agreement with the numerical simulation results, which proves the validity of the numerical simulation results. Through the results of the numerical simulation, the influence of the welding sequence and interlayer temperature on the temperature and residual stress distribution at different locations of the saddle-shaped weld was studied. The results show that the temperature and residual stress distribution on the header and weldolet are asymmetric, and the high-stress area of the saddle-shaped welded joint always appears at the saddle shoulder or saddle belly position. When the interlayer temperature is 300 °C, the peak residual stress reaches a minimum of 428.35 MPa. Adjusting the welding sequence can change the distribution trend of residual stress. There is no high-stress area on the first welding side of the two-stage welding path-2. The peak values of residual stresses for continuous welding path-1 and two-stage welding path-2 are 428.35 MPa and 434.01 MPa, respectively, which are very close to each other.

## 1. Introduction

With the continuous expansion of installed capacity in the thermal power generation industry, the operating parameters of power station boilers have also increased. The increase in boiler temperature and pressure has led to different degrees of damage to pressure-bearing components. The fillet weld of the header pipe works under harsh conditions, and weld cracking often occurs, which leads to leakage accidents and seriously affects the normal operation of generator set [1,2,3,4]. Welding is a complex process involving arc physics, heat and mass transfer, metallurgy, and mechanics. During the welding process, the vicinity of the weld is unevenly heated and cooled by the heat source, resulting in uneven thermal expansion and contraction of the workpiece, which will inevitably lead to the residual stress and deformation of the welded component [5,6,7]. At the same time, the weldolet-header connection form is a grooved butt connection, which is also called a placement type or a saddle-type connection. The welding process of this weld is difficult, and the stress distribution is also more complicated. The saddle shoulder and saddle belly are prone to stress concentration, so welding deformation and cracks are prone to occur [8,9,10,11]. Therefore, how to effectively control the post-weld residual stress and deformation of the header-weldolet welding joint to ensure the safety and reliability of the header work is the primary problem to be solved. Previously, a number of welding scholars conducted detailed research on the residual stress and welding deformation in thick plate butt welding [12,13,14,15], pipe hoop and longitudinal welds [16,17,18], and dissimilar steel welding [19,20,21]. For example, Zhu et al. [22] found that regardless of the welding sequence used, once the base layer, transition layer, and flying edge weld layer were all completed, the highest residual stress concentration was generated in the translation layer and its surrounding area. Wang et al. [23] found that the welding sequence had relatively little effect on the residual stress distribution in the Q345 steel H-joint flange, but there was an effect on the residual stress distribution in the web. Ahmad et al. [24] reduced the interlaminar temperature to the acceptable limit of Al 2219 by establishing an interlaminar cooling process and used experimental measurements of temperature and residual stress to validate the numerical model. Satish et al. [25] found that significant differences in the three-dimensional temperature distribution and residual stress state in AISI304L stub T-joints with different tube sizes along different cross sections. Carrizalez-Vazquez et al. [26] used laser welding of butt joints of DP600 steel plates and reduced the deformation and residual stresses in the specimens by adjusting the welding sequence. Wang et al. [27] found that a slower welding speed and Case B welding sequence resulted in more symmetrical welding. Yi et al. [28] compared the residual stress and deformation after welding by setting up four welding sequences and finally came up with the best welding sequence suitable for the welding of 6061-T6 aluminum alloy automobile bumpers. Hemmatzadeh et al. [29] showed that the interaction of high level of heat input and low level of radius to thickness ratio increased the interlayer temperature. Jiang et al. [30] showed that as the heat input and the number of layers welded increases, the residual stress decreases and the use of multi-layer welding and higher heat input is beneficial in reducing the residual stress. Schroepfer et al. [31] showed that the interlayer temperature has a significant effect on the overall reaction force and that increased heat input and higher interlayer temperature leads to higher strength. Yang et al. [32] showed that within a certain range, increasing the welding interlayer temperature of P91 steel can expand the Mises low stress zone, increase the longitudinal residual compressive stress, and reduce the transverse residual tensile stress, and the maximum interlayer temperature should not exceed 315 °C. Most welding scholars mainly research objects for flat butt joints, T-shaped joints, pipe butt weld joints, and other relatively regular welded joints, but the more complex saddle-shaped welded joints for the weldolet of the multi-layer multi-pass welding structure are rarely researched. The saddle-shaped weld is a spatial curve with changing curvature, the bevel angle of the weldolet is in a state of constant change, and the volume of the weld filler metal material varies with the bevel angle to have the most value. At the same time, the thick-walled weldolet welding requirements in this study are multi-layer multi-pass welding. For a multi-layer multi-pass welding structure, the interlayer temperature between each layer of the weld and the welding sequence between the weld has a very large impact on the quality of the welded joint. The previous research scholars on the butt welding of T-shaped pipes are mainly single-layer welding of thin-walled pipes, while there are few studies on the multi-layer and multi-pass welding structure of thick-walled weldolet saddle welds under different welding process parameters. Therefore, by adjusting the interlayer temperature and welding sequence between each layer of the welds, this paper studies and analyzes the transient temperature field, stress field, and residual stress after welding of the saddle-shaped welded joint of the thick-walled weldolet.

In this paper, welding experiments were performed on the weldolet-header saddle-shaped welded joints. Furthermore, a three-dimensional sequential coupled thermal–metallurgical–mechanical analysis method was established to numerically simulate and analyze the saddle-shaped spatially curved multi-layer multi-pass welding process, and the results obtained were compared with the experimental measurements to verify the reliability of the numerical welding simulation method. Based on the numerical simulation results of the finite element model, the effects of six different welding process parameters on the temperature distribution and residual stress changes at different positions of the weldolet-header saddle weld are compared and analyzed. Finally, the best welding process parameters suitable for multi-layer and multi-pass welding structures of thick-walled welds are obtained, which can provide theoretical guidance for the welding process in actual engineering.

## 2. Experimental Procedure

**Figure 1:**
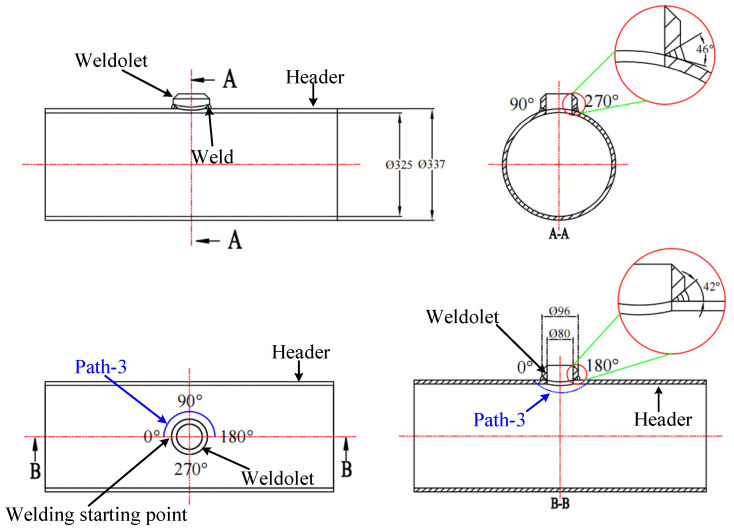
Two-dimensional (2D) schematic diagram of the weldolet-header structure.

**Figure 2:**
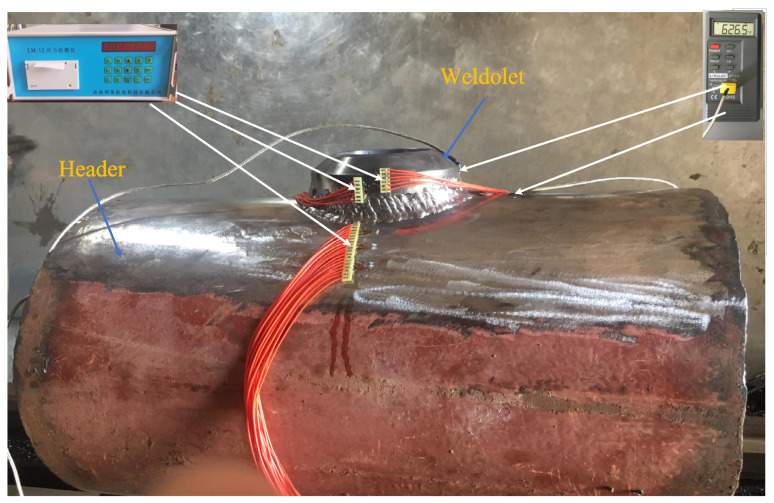
Experiment weld set-up for the weldolet-header.

The size of the header for the welding experiment is φ325 mm × 12 mm × 500 mm, and the size of the weldolet is DN80mm×16mm. The two are vertically welded together to form a T-shaped joint, which can also be called a saddle-shaped welded joint. Figure 1 is a 2D schematic diagram of the weldolet-header structure. The weldolet is a standard part with a groove, and the groove angle varies between 42° and 46°. Header barrel openings [33,34] are without grooves, blunt edges, or and groove gaps. Before welding, sandpaper was used to polish 30 mm around the opening and cleaned with acetone. The welding experiments of the weldolet-header were performed by continuous welding. The current, voltage, and welding speed are shown in Table 1. The welding sequence adopts continuous welding, and interlayer temperature is controlled at 300 °C. Figure 2 shows the schematic diagram of the welding experiment of the weldolet-header. During the welding experiment, the header is placed on the U-shaped base smoothly to ensure the stability of the header. Before welding, four points in the symmetrical direction of the contact line between the weldolet and the header are spot welded firstly, and the purpose of spot welding is to ensure that the line contact between the weldolet and the header is always maintained during the welding process. The base material is 12Cr1MoV, which is a low-alloy pearlite heat-resistant steel. The three welding seams are continuously welded around a circle, using manual tungsten arc welding (TIG) for priming pearlite heat-resistant steel and electrode arc welding (SMAW) for filler and cover welding. Welding material selection is as follows: TIG selects H08CrMoVA wire, φ2.5 mm; SMAW selects R317 welding rod, φ4 mm. Preheating selects the oxygen–acetylene flame gun heating method and uses a thermocouple to measure the temperature. During the whole welding process, we ensured the interlayer temperature was not lower than the preheating temperature.

## 3. Numerical Simulation

Using Simufact Welding 6.0.0 software, the thermodynamic sequence coupling method is used to conduct a thermodynamic analysis of the heat-resistant steel 12Cr1MoV saddle-shaped weld multi-layer and multi-bead welding process. The welding simulation object of this research is a multi-layer multi-pass welding structure. The welding path of each layer of weld is a spatial curve with different curvatures. As the welding heat source moves, the weld material is sequentially filled into the weld seam. Using the “living and dead elements method”, the elements in the area where the heat source is located are activated, and the weld elements in the area where the heat source does not reach are in an inactive state. In the actual welding experiment, the heating time of the oxygen–acetylene flame gun is controlled to preheat and control the interlayer temperature of the weld. In order to simulate this process, part of the base material heated by the flame gun is added with a convective heat transfer boundary condition whose ambient temperature is the interlayer temperature.

### 3.1. 3D Modeling and Meshing

**Figure 3:**
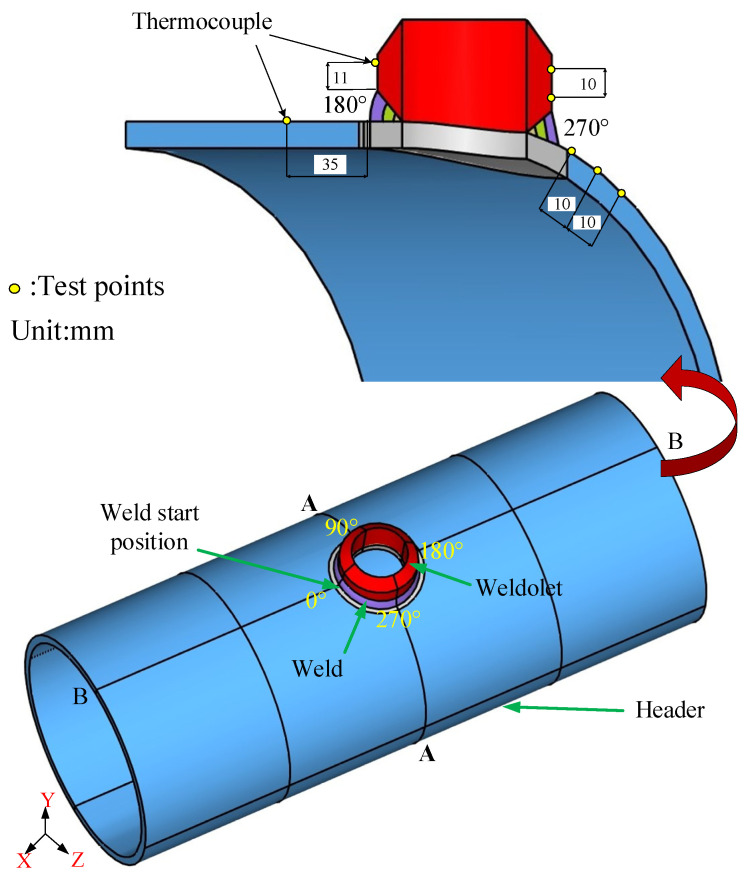
Three-dimensional (3D) model of header, weldolet, and welds.

**Figure 4:**
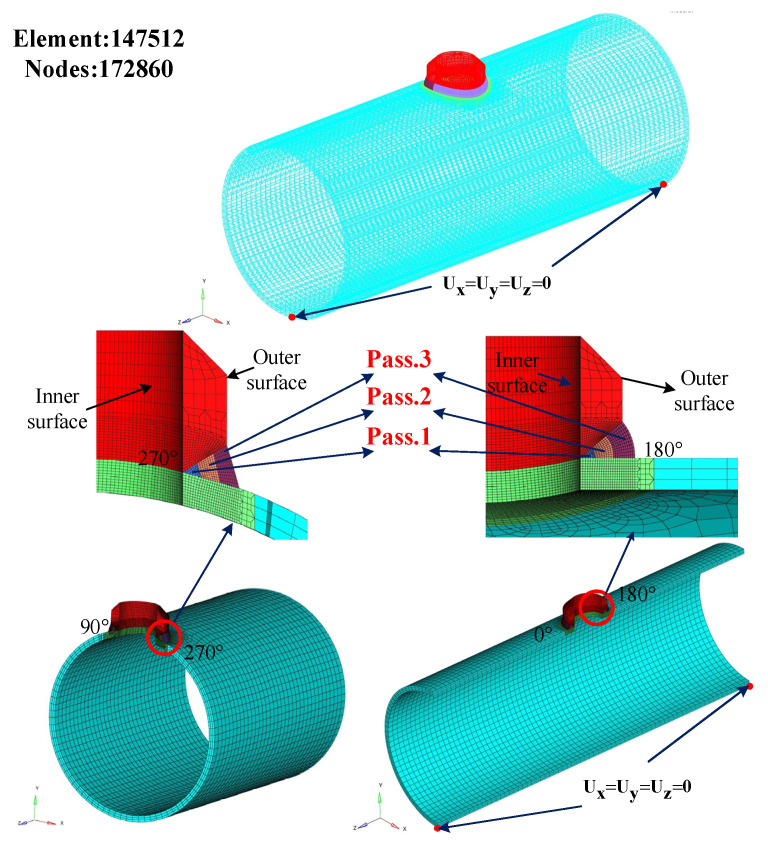
Three-dimensional (3D) finite element mesh with boundary conditions.

In this paper, a 3D model of the weldolet and header is established, which is exactly the same as the actual weldment, as shown in Figure 3. After the model is established, the meshing is performed in Hypermesh meshing software. The structure of this model is symmetrical, so the solid model is divided along the B-B section, and half of the solid model is selected for meshing. Firstly, 2D meshing is performed on the divided section. The mesh type is chosen as eight-node hexahedral element, and a non-uniform mesh is used to take into account the speed and accuracy of the numerical simulation. A transition zone is set between the grids of different regions, and the grids are over-connected to ensure the continuity of the grids. Finally, we used the solidmap command to perform hexahedral meshing. The meshing of the finite element model of the weldolet-header saddle welded joint is shown in Figure 4.

### 3.2. Heat Source Model and Thermal Analysis

Welding temperature field analysis is a typical nonlinear transient heat conduction problem. According to the Fourier heat transfer theorem and the law of conservation of energy, a nonlinear transient heat conduction control equation is established. The temperature field in the welding process varies with time and space, and the transient temperature field T (x, y, z, t) can be expressed by the following Equation (1):

(1) $$\rho c\frac{\partial T}{\partial_{t}}=\frac{\partial }{\partial_{x}}(\lambda \frac{\partial T}{\partial x})+\frac{\partial }{\partial y}(\lambda \frac{\partial T}{\partial y})+\frac{\partial }{\partial_{z}}(\lambda \frac{\partial T}{\partial_{z}})+\;\bar{Q}$$

where ρ is the density of the material; c is the specific heat capacity; T is the temperature; λ is the thermal conductivity; and $\;\bar{Q}$ is the amount of heat generated or consumed per unit of time.

**Figure 5:**
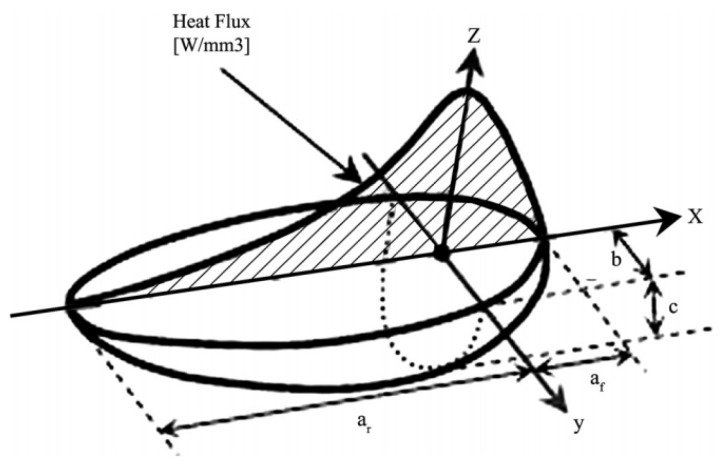
Double ellipsoidal heat source model.

This study uses the double ellipsoid heat source model proposed by Goldak et al. [35]. The specific parameters of the heat source model are obtained by measuring the weld profile size of the welding experiment. The calibrated heat source model is loaded into different welding trajectories to further simulate changes in the thermal field and stress field. The geometry of the double ellipsoid heat source model includes two ellipsoid quadrants. The heat flux distribution function in the front quadrant ellipsoid is $q_{f}\;\left(x,y,z\right)$, and the heat flux density distribution function in the back quadrant ellipsoid is $q_{r}\;\left(x,y,z\right)$. They can be expressed by the following mathematical Equations (2) and (3):

(2) $$q_{f}\left(x,y,z\right)=\frac{6\sqrt{3}f_{f}Q}{{\mathrm{abc}}_{f}\pi \sqrt{\pi }}\exp\left(-\frac{3x^{2}}{c_{f}^{2}}-\frac{3y^{2}}{b^{2}}-\frac{3z^{2}}{c^{2}}\right)$$

(3) $$q_{r}\left(x,y,z\right)=\frac{6\sqrt{3}f_{r}Q}{{\mathrm{abc}}_{r}\pi \sqrt{\pi }}\exp\left(-\frac{3x^{2}}{c_{r}^{2}}-\frac{3y^{2}}{b^{2}}-\frac{3z^{2}}{c^{2}}\right)$$

where Q=ηUI, Q is the energy input rate, U is the arc voltage, I is the welding current, and η is the welding thermal efficiency. The heat loss during welding is inevitable, so η=0.85. The double ellipsoidal heat source model is shown in Figure 5. b is the width of the heat source, c is the depth of the heat source, $a_{f}$ is the length of the front ellipsoid, $a_{r}$ is the length of the back ellipsoid, $f_{f}$ and $f_{r}$ are the front and back ellipsoidal energy distribution coefficients, and $f_{f}+f_{r}=2$. These geometric parameters can be obtained from the weld experimental micrographs. The Gaussian parameters of the double ellipsoid heat source after the verification are shown in Table 2.

**Figure 6:**
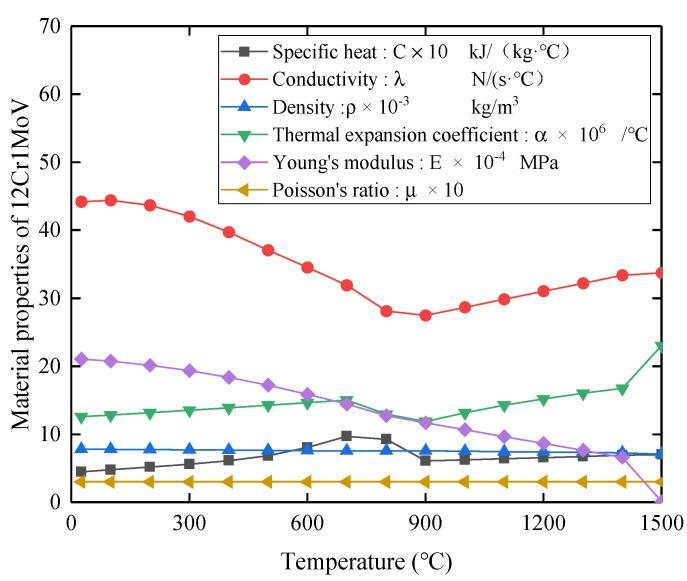
Thermal and mechanical properties of 12Cr1MoV.

In the numerical simulation of welding, the temperature-related thermal and mechanical properties of 12Cr1MoV are considered. The weld filler metal materials H08CrMoVA, R317, and the base material 12Cr1MoV have little difference in performance. Therefore, in the welding simulation process, the performance parameters of the weld filler metal material and the base metal are set to be the same. The thermomechanical property parameters of 12Cr1MoV are shown in Figure 6.

The heated surface of the welded structure will suffer heat loss due to convection and radiation. Convective heat transfer depends on the surface temperature T, the ambient temperature $T_{0}$, and the convective heat transfer coefficient h. The energy loss $Q_{c}$ due to convection can be expressed by the following mathematical Equation (4)

(4) $$Q_{c}=h\left(T-T_{0}\right)$$

The heat loss $Q_{r}$ caused by radiation is calculated according to the Steffen–Boltzmann law, and the mathematical equation is expressed as follows (5)

(5) $$Q_{r}=\varepsilon \sigma \left(T^{4}-T_{0}^{4}\right)$$

where $T_{0}=25{}^{\circ}$ is the ambient temperature, $\sigma =5.67\times 10^{-8}\;W/m^{2}\cdot k^{4}$ is the Steffen–Boltzmann constant,$\;h=20\;W/\left(m^{2}\cdot K\right)$ is the convective heat transfer coefficient, and ε=0.6 is the radiative heat transfer coefficient.

### 3.3. Mechanical Analysis

During the mechanical analysis, the total strain $\varepsilon^{\mathrm{total}}$ of the metal can be defined as several components, and it is described by the following control, as shown in Equation (6):

(6) $$\varepsilon^{\mathrm{total}}=\varepsilon^{e}+\varepsilon^{p}+\varepsilon^{\mathrm{th}}+\varepsilon^{\mathrm{tr}}.$$

In Equation (6), $\varepsilon^{e},{\;\varepsilon }^{p},{\;\varepsilon }^{\mathrm{th}},{\;\mathrm{and}\;\varepsilon }^{\mathrm{tr}}$ represent elastic, plastic, thermo-metallurgical, and phase transformation strains, respectively. For high-carbon steel, the solid–solid phase transformation has a considerable influence on mechanical behavior, and the strain caused by phase transformation should be considered [36]. In addition, the creep-induced strain can also be neglected for the total strain due to the short thermal cycle during the welding process. The elastic strain component is modeled using the temperature-dependent Young’s modulus and Poisson’s ratio of the isotropic Hooke’s law. The plastic behavior adopts the von Mises criterion, temperature-dependent mechanical properties, and an isotropic hardening model. The thermo-metallurgical strain takes into account the temperature-dependent coefficient of thermal expansion and the strain caused by phase change. In the welding experiments, both the weldolet and the header are not constrained. During the numerical simulation, the very minimum boundary conditions are used as artificial boundary conditions to prevent rigid body motion and match the experimental boundary conditions. The boundary conditions are shown in Figure 4, and Ux=Uy=Uz=0 only act on the two nodes at the two ends of the header pipe.

### 3.4. Simulation Cases

**Figure 7:**
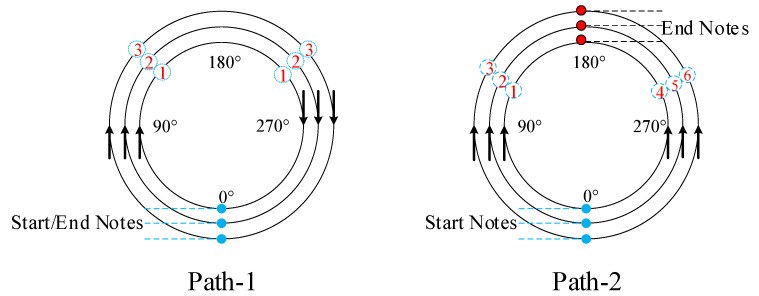
Two welding sequences.

In order to investigate the influence of different welding processes on the temperature distribution and residual stress changes of saddle-shaped welded joints, two different welding sequences were proposed in the numerical simulation process. The details of the welding sequence are shown in Figure 7. Welding path-1 is three welds in a clockwise direction and continuous welding, and the 0° position is both the arc starting point and the arc ending point. Welding path-2 is a two-stage weld, where all processes (processes 1 to 3) on the first side are completed before welding on the second side (processes 4 to 6). The 0° position is the arc starting point of path-2, and the 180° position is the arc ending point of path-2. The arrow represents the welding direction and the number represents the welding order.

At the same time, in order to analyze the influence of the welding interlayer temperature on the temperature field and stress field of the 12Cr1MoV heat-resistant steel saddle-shaped welding joint, three sets of interlayer temperatures were set up on the basis of the above two welding sequences for welding simulation calculation. The welding cases are shown in Table 3, and all other welding parameters of the welding cases are referred to Table 1.

## 4. Experimental Validation of the Numerical Simulation Model

**Figure 8:**
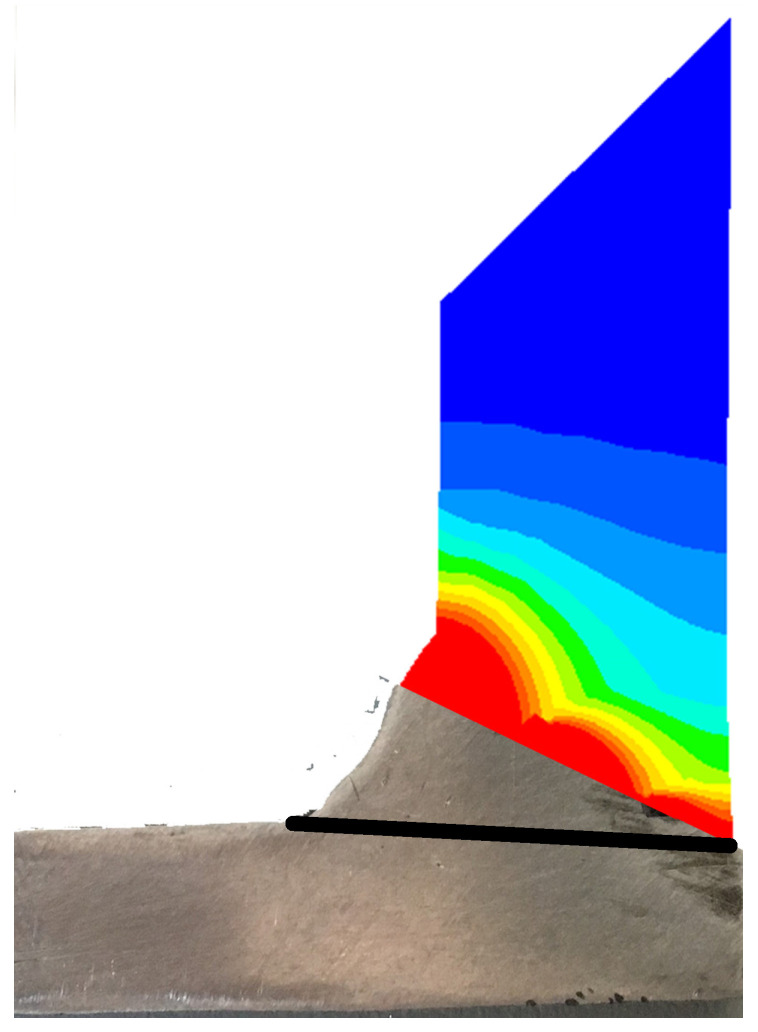
The weld pool shapes obtained by experiments and FE simulation.

In order to verify the rationality of the finite element model, the finite element model in this paper needs to be verified by experiment before welding numerical simulation. In this paper, a welding experimental is carried out on the welding parameters under Case3, and the numerical simulation results are compared with the experimental measurement results. The shape of the experimental weld pool and the predicted weld pool are shown in Figure 8; it can be seen that the pool presents a “funnel shape”. The adjusted heat source parameters match well with the results of the welding experiments, and the finite element model’s melt pool size and shape are consistent with the actual welding pool.

The transient temperature at different locations on the outer surface of the saddle-shaped welded joint was measured using a k-type thermocouple, and the transient temperature was recorded at 2.5 s intervals. The distribution of thermocouples on the outer surface of the header and the weldolet is shown in Figure 3. They are located 35 mm from the center of the weld on the header and 11 mm from the center line of the weld on the weldolet, respectively. The blind hole method was used to measure the post-weld residual stress in the saddle-shaped welded joint, and the location of the residual stress measurement points are shown in Figure 3.

**Figure 9:**
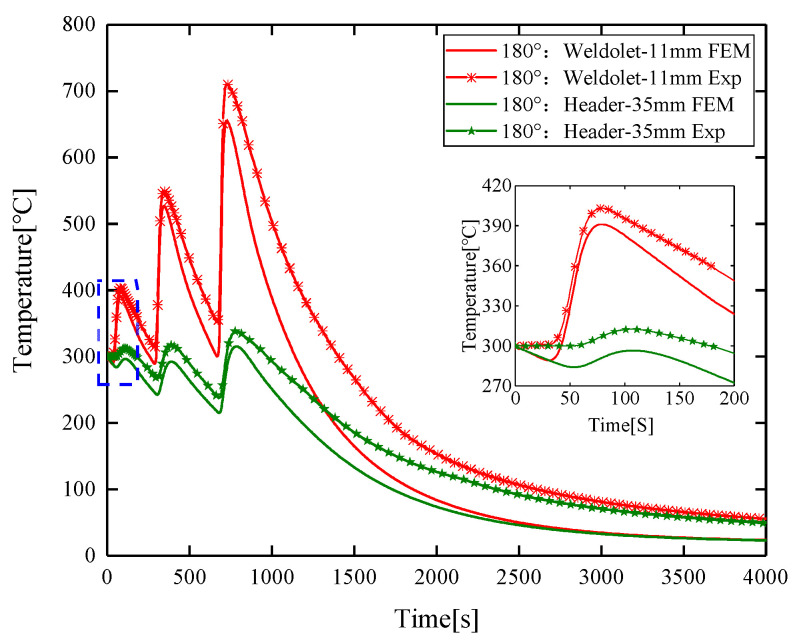
Comparison of the predicted and measured values of transient temperature changes on the outer surface of the weldolet and header.

It can be seen from Figure 9 that the experimental values at 11 mm and 35 mm from the center of the weld are slightly higher than the simulated values at a 180° cross-section, with a maximum difference of 10.03% between the experimental results and the numerical simulation results. Throughout the welding process, the trend of the predicted and measured values remained consistent. When in the heating state, the temperature growth rates remain synchronized. However, in the cooling state, the temperature drop rate of the numerical simulation value is slightly higher than the experimental value temperature drop rate. At the same time, when selecting and zooming in the transient thermal cycle diagram at 0–50 s, it can be seen that the transient thermal cycle in the experiment rises rapidly after being kept at the preheating temperature of 300 °C for a period of time, while the simulation result shows a slight downward trend and then rises rapidly. The reason for this difference may be that the ambient temperature and heat loss of the components in the numerical simulation process are different from the actual welding process.

**Figure 10:**
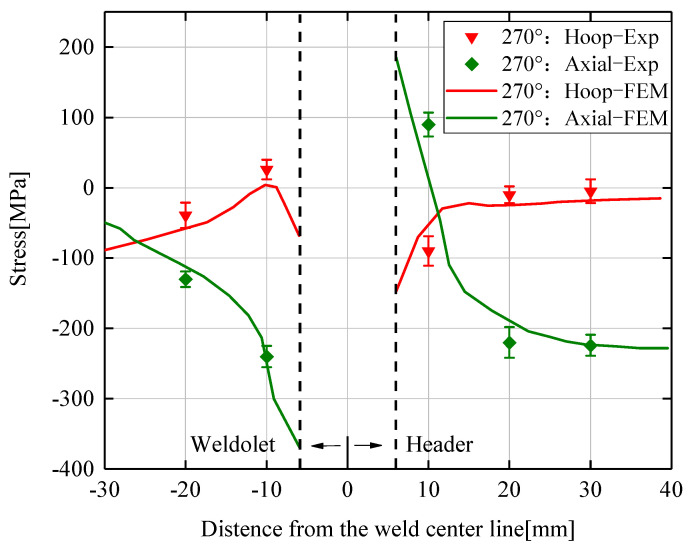
Comparison of experimental results and numerical simulation results of residual stresses.

Five different positions are selected on the outer surface of the 270° cross-section on the weldolet and header to measure the residual stress. The specific position distribution of the measurement points is shown in Figure 3. The numerical simulation results of the hoop and axial residual stresses are plotted in Figure 10 together with the experimental results, with error lines of the experimental data. The abscissas on the left and right sides of the origin of the coordinate system respectively represent the residual stresses on the outer surface of the weldolet and the header. The residual stress curve at the weld seam in the figure is discontinuous, and the residual stress at the weld seam is not shown in the numerical simulation results for the reasons described in Section 5.2. It can be seen from Figure 10 that the axial and hoop residual pressure predicted by the finite element model are consistent with the experimental results, but there is a certain difference. The reason for this difference may be the inaccuracy of the finite element model or the error of the measurement results. Another reason is that there are residual stresses caused by manufacturing and processing inside the header and the weldolet. In the welding process, the residual stress generated in the manufacturing process will indirectly combine with the stress generated in the welding process, and ultimately affect the distribution trend of the residual stress. The finite element model ignores the residual stresses generated by the manufacturing process. Based on the above analysis, it is shown that the numerical simulation results and the experimental results are consistent. The experimental validation of the established finite element model was also completed.

## 5. Results and Discussion

### 5.1. Analysis of Welding Temperature Field Results

**Figure 11:**
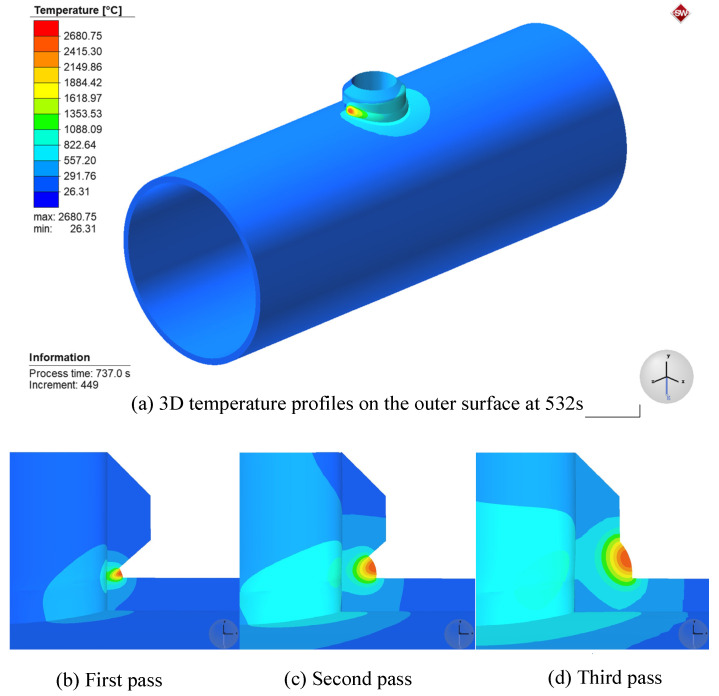
Three-dimensional (3D) temperature profiles.

Figure 11a shows the 3D temperature profile of the outer surface at the moment of 532 s under the Case3 condition in Table 3. It can be seen from the figure that the temperature in the center of the weld is the highest, reaching 2198.3 °C, which is higher than the melting point of the base material 12Cr1MoV at 1520 °C. The temperature spreads from the center of the weld to the surroundings in a wave shape. The instantaneous temperature change in front of the melt pool is fast, while the instantaneous temperature change at the back of the melt pool is relatively slow, and the isotherm shows a trailing shape. Figure 11b–d show the 3D temperature profiles of the different layers of the weld. The molten pool of the first weld has a smaller volume and requires less filler metal. As the bevel angle increases, the volume of the melt pool expands, so the area of the heat-affected zone also gradually expands.

**Figure 12:**
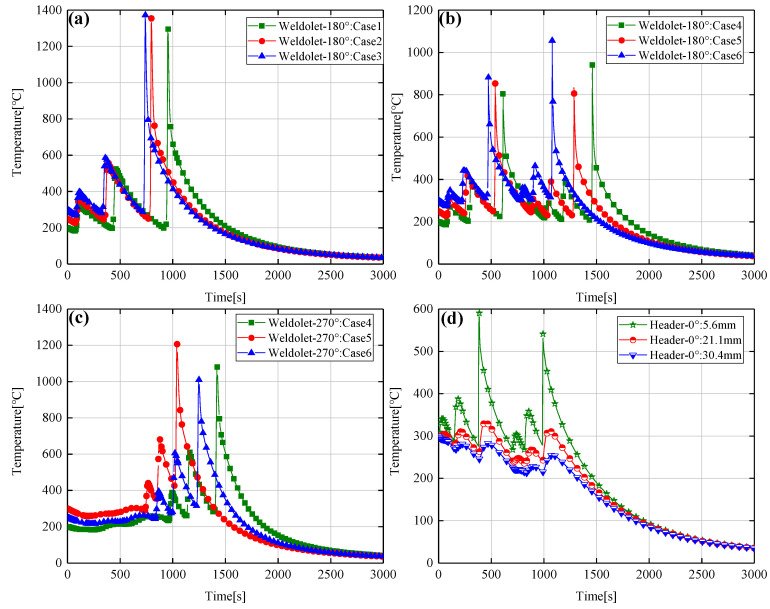
(**a**) Transient thermal cycling of the 180° section path-1, (**b**) Transient thermal cycling of the 180° section path-2, (**c**) Transient thermal cycling of the 270° section path-2, (**d**) Transient thermal cycling of the 0° section at 5.6 mm, 21.1 mm, 30.4 mm from the center of the weld.

Figure 12a–d shows the temperature distribution of the saddle-shaped welded joints at 0°, 180°, and 270° sections under six different welding processes (Case1–Case6). Figure 12a shows the transient temperature distribution of different interlayer temperatures at path-1. Each thermal cycle curve has three peaks and three troughs, representing the highest and lowest temperatures of the weld, respectively. The peak temperatures of the first two peaks are not very different, while the peak temperature of the third peak is much higher than that of the first two peaks, which is related to the distance from the center of the heat source. Figure 12b shows the transient temperature distribution of different interlayer temperatures at path-2. It can be seen that the transient temperature distribution at the 180° cross-section of path-2 is more complex than that of path-1, with six peaks. This is because the 180° position is the arc ending point of path-2, not the arc ending point of path-1.

Figure 12c shows the transient temperature distribution of path-2 at the 270° section. It can be seen that the temperature profile fluctuates very little from 0 to 800 s, which is due to the fact that 270° section belongs to the second side of path-2 and receives little thermal influence from the first side. The welding method of path-2 and path-1 at the 270° section is the same, so there are only three peak temperatures. Figure 12d shows the transient temperature of path-2 at different distance points from the center of the weld at the 0° (at the arc starting position) cross-section of the header. It can be seen that the transient temperature at 0° and 180° of path-2 has the same pattern of variation, and the peak temperature value of transient temperature decreases as the distance from the center of the weld increases. By comparing the transient temperature distribution at the same distance point from the weld on the header and the weldolet, it can be found that the peak temperature of the point taken on the weldolet is higher than the temperature of the point taken on the header. This is because the wall thickness and diameter of the weldolet and the header are very different, the temperature conduction rate on the weldolet is faster. It can also be seen from Figure 11 that the area of the heat-affected zone on the weldolet is larger.

### 5.2. Analysis of Welding Stress Field Results

**Figure 13:**
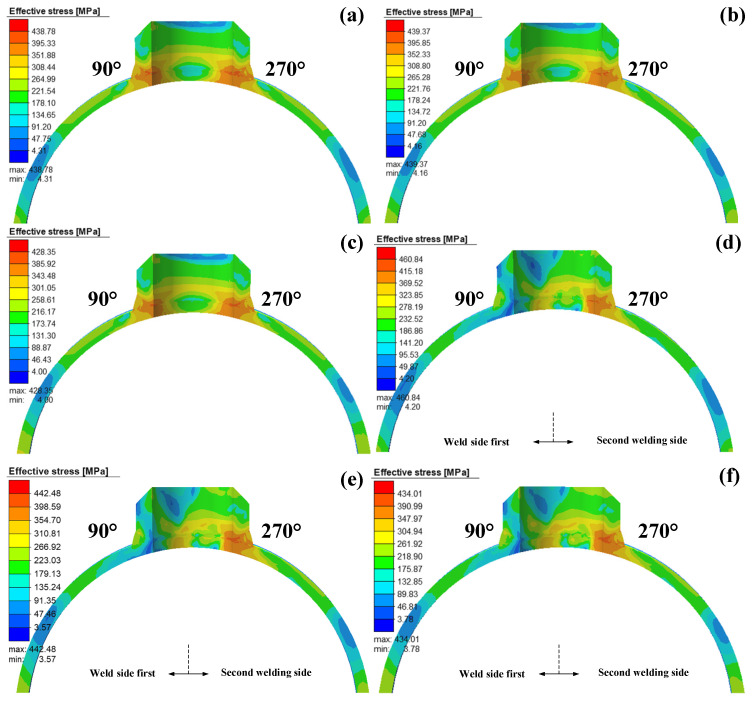
Contours of Von Mises stress for the 90°–270° cross-section: (**a**) Case1, (**b**) Case2, (**c**) Case3, (**d**) Case4, (**e**) Case5, (**f**) Case6.

Figure 13 shows the Von Mises stress distribution for Case 1–Case 6 at the 90°–270° cross-section. The Von Mises stress diagram shown in the figure is the 4000 s welding time, that is, the Von Mises stress when the weld is cooled to room temperature. It can be seen that the Von Mises stresses distribution of Case 1–Case 3 (welding path-1) is symmetrical on the cross-section, with a large high-stress zone area appearing at the saddle belly (at 90°and 270° positions). By comparing and observing the color legend of the post-processing window, it can be seen that as the interlayer temperature increases, the maximum stress decreases. It can also be seen that the Von Mises stress value at the saddle shoulder (0° and 180° positions) of the saddle weld under path-1 is about half that of the saddle belly. This is because the weld has the greatest curvature at the saddle web position and the bevel angle is at its maximum. The greater the bevel angle, the more the filler metal, the more obvious the effect of metal heating expansion and cooling shrinkage during the welding process. Meanwhile, the area of the high-stress zone in the 270° section in Case1–Case3 is slightly larger than that in the 90° section, which is due to the thermal effect of the weld heat generated in the 270° section on the weld in the 90° section, reducing the residual stress inside the weld.

In Case 4–Case 6 (welding path-2), the effect of interlayer temperature on stresses follows the same pattern as in path-1. However, the Von Mises stresses of path-2 is asymmetrically distributed on the cross-section. The high-stress areas in the first weld area disappear, while the high-stress areas in the second weld side still exist. It can also be seen that the Von Mises stress distribution at the saddle shoulder of path-2 is more complex than that of patn-1, and the stress values have also increased. Therefore, a reasonable adjustment of the welding sequence can effectively reduce the Von Mises stress inside the weld, which is of great significance to the special welding characteristics of the saddle-shaped welded joint.

In order to further investigate the influence of welding sequence on the residual stress at different positions of the saddle-shaped weld, Case3 and Case6 in the cases are selected for analysis. The residual stress distribution at the 0°, 90°, 180° and 270° sections of the saddle-shaped weld under two welding sequences are compared and analyzed. The saddle-shaped welded joints belong to T-shaped pipe joints. The residual stress at the weld cannot be determined whether it is related to the header or the weldolet, so the direction of the stress cannot be defined. For example, it can be seen from Figure 2 that the stress components along the y-axis direction represent the hoop stress in the header and the axial stress in the weldolet. Therefore, the residual stresses at the weld seam are not showed in the figures.

**Figure 14:**
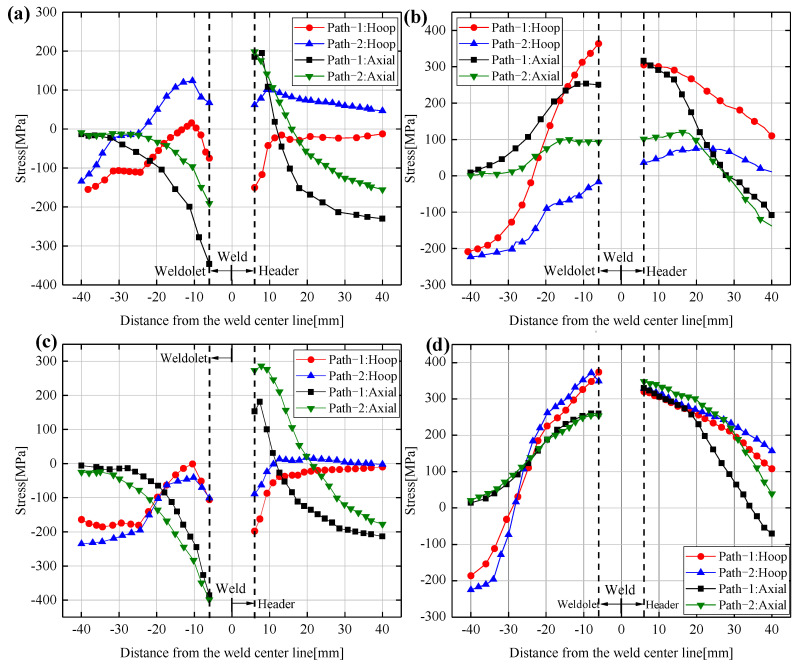
Hoop and axial residual stress distribution for Case3 and Case6 at the 90° and 270° sections: (**a**) Outer surface of the 90° section, (**b**) inner surface of the 90° section, (**c**) outer surface of the 270° section, (**d**) inner surface of the 270° section.

Figure 14a–d show the distribution of the hoop and axial residual stresses on the inner and outer surfaces of the 90° and 270° sections for the two welding sequences, respectively. It can be seen that the residual stress distribution trend of path-1 is almost the same on the two sections. The hoop residual stresses on the outer surface of welding path-1 are mainly compressive stresses. While the inner surface is tensile stress near the weld seam and compressive stress away from the weld seam. The maximum value of axial residual stress on the outer surface of the welding path-1 occurs near the weld seam, and the axial residual stress is distributed in the opposite direction on the outer surface of the weldolet and the header. The axial residual stress on the inner surface of path-1 has the same distribution trend on the weldolet and header on both sides of the weld. The difference between the hoop and axial residual stresses on the outer surfaces of path-2 at 90° and 270° sections mainly reflects the maximum value of residual stresses. The maximum value of residual stresses at 270° section is larger than that at 90° section, and path-2 has much higher hoop and axial residual stresses on the inner surface of 270° section than 90° section.

The hoop and axial residual stress distribution trends of the two welding sequences are more similar in the 270° section. The residual stress distribution of welding path-1 in the two cross-sections is more symmetrical, while the residual stress of welding path-2 in the 90° cross-section is significantly lower. Overall, welding path-2 has less residual stress than path-1 in both sections.

**Figure 15:**
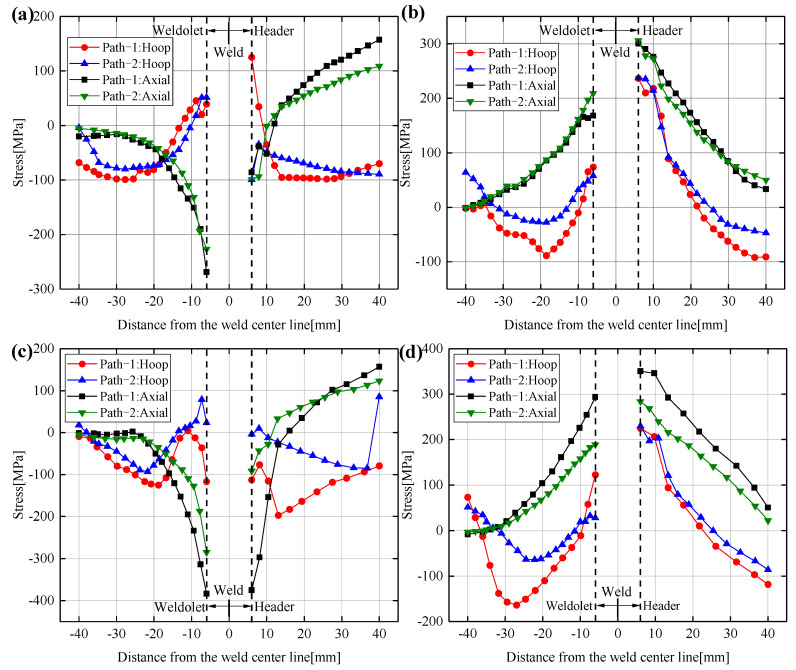
Hoop and axial residual stress distribution of Case3 and Case6 at the 0° and 180° sections: (**a**) Outer surface of the 0° section, (**b**) Inner surface of the 0° section, (**c**) Outer surface of the 180° section, (**d**) Inner surface of the 180° section.

The 0° position is the arc starting and ending point of path-1 as well as the arc starting point of path-2. Figure 15a–d show the distribution of the hoop and axial residual stresses on the inner and outer surfaces of the 0° and 180° sections for the two welding sequences, respectively. It can be seen that the difference in the distribution of residual stresses between the 0° and 180° sections is mainly in path-1. The residual stresses of path-1 at the 0° section are less than those in the 180° section. This is due to the fact that the 0° position has experienced more thermal cycles and the residual internal stresses inside the weld has reduced. The hoop residual stress distribution trend of the welding path-1 on the outer surface of the 180° section is similar to the distribution trend of the 90° and 270° sections, mainly compressive stress. The residual stress distributions of path-2 at the 0° and 180° sections are very similar, because the number of thermal cycles experienced is the same. It can also be seen that the residual stresses on the inner surfaces of path-2 at 0°and 180° sections are smaller than those of path-1.

There is no uniform law for the distribution of the hoop residual stresses on the inner and outer surfaces of the weldolet and the header. Meanwhile, the axial residual stresses on the outer surfaces of the weldolet and the header are inversely symmetric and the axial residual stresses on the inner surfaces are positively symmetric. The axial residual stresses on the weldolet are high near the weld seam, and the axial residual stresses tend to 0 MPa at the end of the weldolet. The locations where the differences in residual stresses are large for path-1 and path-2 are the 90° and 180° sections, while the differences in residual stress distribution are relatively small at the 0° and 270° sections.

**Figure 16:**
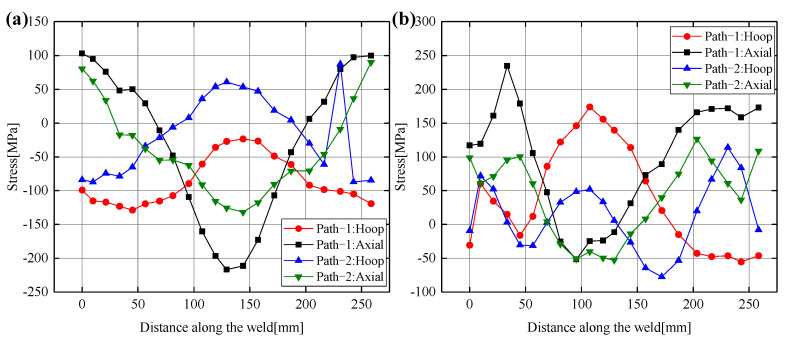
Hoop and axial residual stress distribution along the weld direction for Case3 and Case6 in the 0°–180° weld path: (**a**) Outer surface residual stress, (**b**) Inner surface residual stress.

Figure 16 shows the residual stress distribution along the weld direction for both welding sequences in the 0°–180° welding path (path-3 was selected on the header as shown in Figure 1). Along the welding path direction, it can be seen that path-1 and path-2 have similar residual stress distribution trends on the outer surface, and the residual stresses are well symmetrical along the weld direction. The residual stresses on the outer surface are very close to each other at the starting point (0° position) and the ending point (180° position) of path-3, and the residual stress values are both relatively large. The residual stress values at the midpoint (90° position) of path-3 are also large. The distribution trends of axial residual stress and hoop residual stress on the outer surface of path-1 and path-2 are opposite. The hoop residual stress distribution presents a peak shape, and the axial residual stress distribution presents a valley shape. However, the axial and hoop residual stresses on the inner surfaces of path-1 and path-2 show several fluctuations. Especially, the fluctuations of the hoop and axial residual stresses in path-2 are more obvious near the 0° and 180° positions. The reason for the fluctuation is the instantaneous temperature change in the process of arc starting and ending for many times. It can also be found that the axial and hoop residual stresses on the inner and outer surfaces of path-1 are always higher than those of path-2, which is consistent with the conclusions drawn above.

## 6. Conclusions

In this paper, through numerical simulation and experimental verification methods, the effects of welding sequence and interlayer temperature on the temperature and residual stress distribution of the weldolet–header saddle welded joint were studied. In different sections and positions, the temperature and residual stress of the inner and outer surfaces of the header and the weldolet are compared and analyzed. Based on the results of this study, the major conclusions drawn are the following:

1. In this paper, the heat source is fitted to the finite element model, and the size of the experimental melt pool matches that of the melt pool in the finite element simulation. The experimentally measured transient thermal cycles and residual stress distribution are in agreement with the numerical simulation results, which proves the validity of the finite element model established.
2. The welding sequence is a key factor affecting the residual stress distribution in welding. The peak residual stress for the continuous welding path-1 is 428.35 MPa and the peak residual stress for the two-stage welding path-2 is 434.01 MPa, which are very close values. The two-stage welding path-2 does not have a high-stress area on the first welding side, so it is more suitable for welding saddle-shaped welding joints.
3. The interlayer temperature of the weld is the main factor affecting the maximum value and distribution area of welding residual stress. In the two-stage welding path-2, when the interlayer temperature increases from 200 to 300 °C, the peak value of the residual stress gradually decreases from 460.84 to 434.01 MPa, and the distribution area of the high-stress area inside the weld is also reduced.
4. Saddle welded joints have special welding characteristics. Although adjusting the welding sequence can change the distribution of residual stresses, high-stress areas always appear at the saddle shoulder (at the 0° and 180° sections) or at the saddle web (at the 90° and 270° sections).
5. The temperature and residual stress distribution on the header and the weldolet are asymmetrical, and the range of the high temperature area on the weldolet is larger than that on the header. The axial residual stresses on the outer surface are inversely distributed on the weldolet and the header, while the distribution trend on the inner surface is symmetrical to some extent. The hoop residual stresses on the inner and outer surfaces are distributed asymmetrically on the weldolet and the header, but the distribution trends are similar.

## Figures and Tables

**Table 1:** Welding parameters.

Welding Pass	Welding Method	Current/A	Voltage/V	Welding Speed/mm·s^−1^
1	TIG	93	18	1.6
2	SMAW	165	25	2
3	SMAW	220	30	2

**Table 2:** Heat source parameters.

Welding Layer	Front Axle Length $a_{f}$/mm	Rear Axle Length $a_{r}$/mm	Width b/mm	Depth d/mm
1	1.2	4.4	2.0	6.2
2	2.4	13.2	6.0	9.8
3	5.0	24.0	13.0	10.0

**Table 3:** Welding cases.

Case	Welding Sequence	Preheating Temperature/°C	Interlayer Temperature /°C
Case1	Path-1	200	200
Case2	Path-1	250	250
Case3	Path-1	300	300
Case4	Path-2	200	200
Case5	Path-2	250	250
Case6	Path-2	300	300

## Data Availability

Not applicable.
